# Feasibility and acceptability of peer-led assessment of HIV risk among female sex workers in Zimbabwe

**DOI:** 10.1136/bmjgh-2024-017968

**Published:** 2025-12-05

**Authors:** Primrose Matambanadzo, Euphemia Lindelwe Sibanda, Richard Steen, James R Hargreaves, Frances M Cowan, Joanna Busza

**Affiliations:** 1 Key Populations, Centre for Sexual Health and HIV/AIDS Research, Harare, Zimbabwe; 2 International Public Health, Liverpool School of Tropical Medicine, Liverpool, UK; 3 CeSHHAR Zimbabwe, Harare, Zimbabwe; 4 London School of Hygiene & Tropical Medicine Faculty of Public Health and Policy, London, UK; 5 Centre for Sexual Health and HIV/AIDS Research, Harare, Zimbabwe; 6 Population Health, London School of Hygiene and Tropical Medicine, London, UK

**Keywords:** Zimbabwe, HIV

## Abstract

**Introduction:**

Female sex workers (FSWs) in sub-Saharan Africa are at higher risk of HIV acquisition and transmission. However, FSWs’ life experiences are heterogeneous and are important to characterise in order to tailor responses to individual needs. Peer microplanners are FSWs trained to engage other FSWs and offer each individual a tailored package of support. We sought to assess the feasibility, acceptability and fidelity of implementing peer-led assessments of risk.

**Methods:**

Between December 2020 and August 2021, we conducted a qualitative process evaluation study of the Adapted Microplanning: Eliminating Transmissible HIV In Sex Transactions (AMETHIST) trial’s peer-led assessment of FSWs’ risk of HIV acquisition and transmission. We enrolled 60 participants (30 FSW and 30 peer microplanners) for semistructured in-depth interviews at 3 of 11 intervention sites. Interview guides and analysis were underpinned by three elements adapted from the UK Medical Research Council’s process evaluation framework: (1) feasibility, (2) acceptability and (3) fidelity. Using a framework analytic approach, data were familiarised, coded with NVivo V.12 software, charted, interpreted and then deductively mapped to these three elements.

**Results:**

Peer microplanners used the risk scores to tailor their support to FSW with good fidelity. Some experienced difficulties completing assessments during the initial period of implementation. However, it was feasible for peer microplanners to establish strong relationships, and they used observational and conversational skills to elicit sensitive information from FSW to support their assessment. The assessments and tailored support offered by peer microplanners were acceptable to FSWs, although some did not appear to know they were being assessed.

**Conclusions:**

Our findings show that peer microplanners feasibly and acceptably assessed FSW for certain risk factors through a scoring tool and focused their support accordingly. This approach could be integrated into other peer-led interventions to improve prioritisation of HIV programmes.

**WHAT IS ALREADY KNOWN ON THIS TOPIC**

**WHAT THIS STUDY ADDS**

- Fills a literature gap by demonstrating that peer-led assessments are feasible, can be implemented with fidelity and are acceptable to FSWs.

**HOW THIS STUDY MIGHT AFFECT RESEARCH, PRACTICE OR POLICY**

- Implementers should consider adapting and scaling up peer-led assessments of HIV risk to other key populations.
- Policy-makers should consider supporting the integration of peer-led assessments of HIV risk into public sector services to improve HIV prevention efforts.

## Introduction

 Although female sex workers (FSWs) make up between 1.3% and 1.6% of the general population, an estimated 15% of new HIV infections in sub-Saharan Africa are among FSWs.^1 2^ FSWs are at heightened risk of HIV acquisition and transmission. However, FSW experiences are heterogeneous and influenced by bio-behavioural and structural factors, with some FSWs more exposed to HIV infection than others.^3^,^7^ Global guidance recommends peer leadership of HIV prevention interventions to effectively address needs and facilitate reduced exposure to HIV risk factors among FSW.^8 9^ To this end, peer microplanners are FSWs trained in enhanced peer outreach to engage other FSWs individually and offer a tailored package of support (health education, condoms and lubricants, linkage to HIV services, etc).^10^,^12^ These well-networked peers map geographical areas where they live and work and gather data about FSWs found in that location using a defined set of tools. They enrol FSWs into their caseloads and use data collected on each individual to plan outreach activities and link each person to services that meet their unique needs.^10^ A growing body of literature on HIV programming with FSW in various settings, including India, Kenya and more recently Zimbabwe, has demonstrated the effectiveness of microplanning in improving reach, linkage to and uptake of services.^210 11 13^,^15^

In addition to peer leadership of interventions, understanding differential risk is important to ensure that the appropriate level of support is given to those who need it most. Assessments of risk have been increasingly prioritised as a gateway to FSWs’ uptake of HIV prevention interventions.^316^,^19^ Several studies have explored tools to assess risk. These have mainly been quantitative checklists administered by researchers or service providers to screen individuals into or out of services.^1720^,^24^ However, opportunities for enhancing engagement in HIV services by those most in need are often missed through misclassification.^17 20^

Assessments of risk of HIV infection have been incorporated into microplanning with FSW in several countries in Africa, usually administered within health facilities by programme staff.^13 14^ Delivery of these remains poorly understood; however, there is potential for peer microplanners’ to use them to more effectively tailor support. Nested within a nationally scaled programme providing services for sex workers in Zimbabwe, the Adapted Microplanning: Eliminating Transmissible HIV In Sex Transactions (AMETHIST) cluster randomised trial used quarterly assessments of risk conducted by peer microplanners to determine the frequency and content of FSWs’ support. A process evaluation of the AMETHIST trial was conducted in three purposively selected sites.

This study aims to assess the feasibility and acceptability of implementing peer-led assessments of HIV risk and whether these were completed with fidelity.^13 25^

## Methods

Between December 2020 and August 2021, we conducted a qualitative process evaluation of peer-led assessment of FSWs’ risk of HIV acquisition and transmission within the AMETHIST trial.

### Intervention description

**Figure 1:**
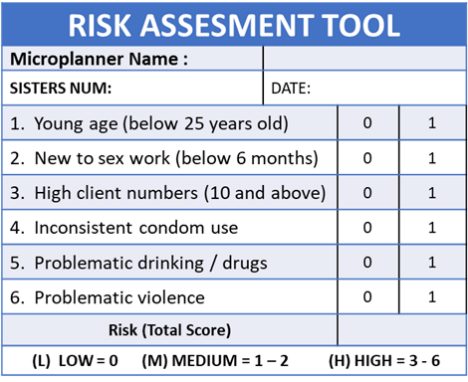
Six-factor risk assessment tool.

The intervention being evaluated recruited and trained 104 peer microplanners. Each was responsible for supporting 50–80 FSW from the mapped geographical areas assigned to them. FSWs were listed in ‘hotspot diaries’ (a caseload record) following verbal consent. Peer support was ‘status neutral’, meaning peer microplanners did not ask about HIV status in order to provide support and promote clinic attendance.’A six-factor AMETHIST risk assessment tool (figure 1) was used to assign FSWs to one of three groups: FSWs at higher risk/FSWs at moderate risk/FSWs at lower risk. The primary purpose was to prioritise individuals for more attention. The AMETHIST risk factors included age, duration of time in sex work, number of clients per week, consistency of condom use, use of alcohol and other substances and experience of violence. The risk factors used were intuitive, had been agreed in consultation with peer microplanners and used in risk categorisation tools elsewhere.^13 26^

As part of their initial 5-day training, peer microplanners were trained to undertake assessments of risk for all FSWs they enrolled into their caseloads. The first assessment was to be conducted after a peer microplanner had established rapport and built a relationship with the individual enrolled in their caseload. Short worksheets (4–5 prompts) were provided to help guide discussions about factors 4, 5 and 6 (online supplemental appendix 1). The peer microplanners were not asked to explicitly explain assessments to FSWs but were not prevented from doing so. Assessment results were not shared with the person concerned but used to guide subsequent support.

**Figure 2:**
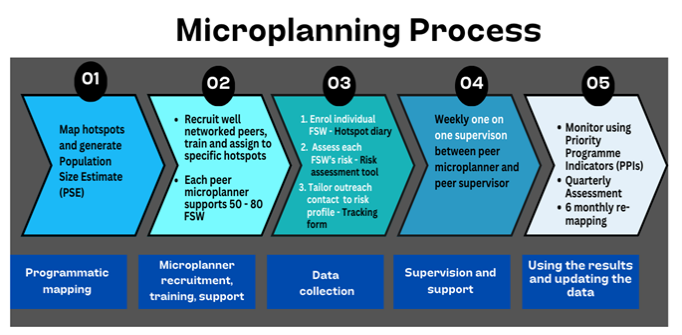
Microplanning processes. FSW, female sex worker.

A completed assessment resulted in a score assigned to each woman reflecting the peer microplanner’s most accurate knowledge of the person’s characteristics, behaviours and experiences. This score determined outreach frequency, ensuring those FSWs at higher risk were seen and supported most often. Figure 2 shows the microplanning process and how the risk group guided frequency of outreach contacts (‘tracking’). FSWs at lower risk were to be seen once a month, those at moderate risk once a fortnight, and FSWs at higher risk were seen once weekly. During each outreach contact, the peer microplanner delivered counselling on various topics most suited to the individual. Peer microplanners also used outreach contacts to supply condoms and lubricants, taking into account expected number of partners, and encouraged FSW to attend clinical services.

To reduce recall and social desirability bias in self-reporting behaviours, peer microplanners were trained to undertake assessments only after building rapport with FSW and to use a mix of direct observations and unstructured discussions to elicit data.^27^ Assessments were to be repeated every 3 months to capture and respond to changes over time.^21^ Peer supervisors were deployed to provide supervision and ongoing motivation to microplanners, support their work planning, help solve local problems and monitor progress towards goals in weekly one-on-one meetings.

### Qualitative study setting

Qualitative data were collected at 3 of the 11 intervention sites in Zimbabwe. These were purposively selected for the process evaluation based on the diversity of geographic and sex worker population characteristics in line with process evaluation guidance from the UK Medical Research Council (MRC). The MRC guidance recommends intervention complexity, potential mechanisms of impact and the varying contexts be reflected in site selection.^28 29^ To support transferability (assessing applicability elsewhere), contextual descriptions and characteristics of the three sites are provided in table 1.^30^

**Table 1:** Characteristics of sites purposively selected for qualitative data collection

Province	District	Name of site	Characteristics	Sex work typologies	Initial population size estimate* (June 2019)	Final population size estimate (May 2021)
Manicaland	Makoni	Rusape	Small periurban town on the main highway to Mozambique	street, bar, shebeen, home-based, online/phone, around army base. Truck stop	**657**	**954**
Masvingo	Chivi	Ngundu	Along a major highway on the way to South Africa	bar, truck stop, street, online/phone	**244**	**347**
Mashonaland West	Makonde	Chinhoyi	Large urban town with a university on the main highway to Zambia	Bar, home-based, street, phone/online, around artisanal mining areas	**479**	**1473**

* Population size estimate = number of FSWs estimated to be present during peak hours within all known ’hotspots’ during mapping, aggregared to generate a localised population size estimate (PSE).

### Participant involvement

Peer microplanners from a pilot site were involved in the design and conduct of this research. Female sex workers were engaged in the community advisory board (CAB) to ensure the intervention design and methods of recruitment reflected the needs and preferences of FSW and that materials were culturally appropriate. The cluster randomised trial results were fed back to the CAB in a manner suitable for a non-specialist audience.

### Sampling of study participants

Semistructured in-depth interviews were conducted with 60 participants: 30 FSWs aged 19–46 years (median age 31 years) and 30 peer microplanners aged 21–50 years (median age 31 years) (see table 2). There were 10 peer microplanners and 10 FSWs recruited at each of the three sites. We sought to capture diverse experiences and perspectives of undertaking microplanning and of being a microplanned FSW by employing a purposive sampling technique. Participants were recruited from the following groups with characteristics that would support theoretical representativeness: FSW who were members of self-help groups (SHG) and those who had not joined any SHG (which were also a component of the AMETHIST intervention), FSW who had accessed services within the programme and those who had not, and FSW who were being microplanned and those who were not. For peer microplanners, we recruited those with a dwindling caseload or sustaining a large caseload, as well as microplanners who had and had not established at least one SHG (which was part of their role). No participants refused to participate or later dropped out of this qualitative study.

**Table 2:** Characteristics of sampled participants

Participants ID	Site	Age	Microplanned (Y/N)	SHG (Y/N)	Participant ID	Site	Age	Caseload size: ≤49/≥50
FSW1	Chinhoyi	>35 years	Y	Y	Peer microplanner 1	Chinhoyi	25–35 years	≥50
FSW2	Chinhoyi	>35 years	Y	Y	Peer microplanner 2	Chinhoyi	25–35 years	≤49
FSW3	Chinhoyi	>35 years	Y	N	Peer microplanner 3	Chinhoyi	25–35 years	≥50
FSW4	Chinhoyi	25–35 years	Y	Y	Peer microplanner 4	Chinhoyi	>35 years	≥50
FSW5	Chinhoyi	25–35 years	Y	N	Peer microplanner 5	Chinhoyi	>35 years	≥50
FSW6	Ngundu	≤24 years	Y	N	Peer microplanner 6	Ngundu	>35 years	≥50
FSW7	Ngundu	25–35 years	Y	Y	Peer microplanner 7	Ngundu	25–35 years	≤49
FSW8	Ngundu	>35 years	Y	Y	Peer microplanner 8	Ngundu	25–35 years	≥50
FSW9	Ngundu	25–35 years	Y	Y	Peer microplanner 9	Ngundu	>35 years	≥50
FSW10	Ngundu	>35 years	Y	Y	Peer microplanner 10	Ngundu	25–35 years	≥50
FSW11	Rusape	≤24 years	Y	Y	Peer microplanner 11	Rusape	25–35 years	≥50
FSW12	Rusape	25–35 years	Y	N	Peer microplanner 12	Rusape	25–35 years	≥50
FSW13	Rusape	>35 years	Y	N	Peer microplanner 13	Rusape	25–35 years	≥50
FSW14	Rusape	25–35 years	Y	N	Peer microplanner 14	Rusape	≤24 years	≤49
FSW15	Rusape	25–35 years	Y	N	Peer microplanner 15	Rusape	>35 years	≤49
FSW16	Chinhoyi	25–35 years	Y	N	Peer microplanner 16	Chinhoyi	>35 years	≤49
FSW17	Chinhoyi	25–35 years	Y	N	Peer microplanner 17	Chinhoyi	>35	≥50
FSW18	Chinhoyi	≤24 years	Y	Y	Peer microplanner 18	Chinhoyi	25–35 years	≥50
FSW19	Chinhoyi	25–35 years	Y	Y	Peer microplanner 19	Chinhoyi	≤24 years	≤49
FSW20	Chinhoyi	25–35 years	Y	Y	Peer microplanner 20	Chinhoyi	25–35 years	≥50
FSW21	Ngundu	25–35 years	Y	Y	Peer microplanner 21	Ngundu	25–35 years	≤49
FSW22	Ngundu	>35 years	Y	Y	Peer microplanner 22	Ngundu	25–35 years	≥50
FSW23	Ngundu	25–35 years	Y	Y	Peer microplanner 23	Ngundu	>35 years	≥50
FSW24	Ngundu	>35 years	Y	Y	Peer microplanner 24	Ngundu	25–35 years	≥50
FSW25	Ngundu	25–35 years	Y	Y	Peer microplanner 25	Ngundu	>35 years	≥50
FSW26	Rusape	25–35 years	Y	N	Peer microplanner 26	Rusape	25–35 years	≤49
FSW27	Rusape	>35 years	Y	Y	Peer microplanner 27	Rusape	≤24 years	≥50
FSW28	Rusape	25–35 years	Y	Y	Peer microplanner 28	Rusape	25–35 years	≥50
FSW29	Rusape	≤24 years	Y	Y	Peer microplanner 29	Rusape	25–35 years	≥50
FSW30	Rusape	>35 years	Y	Y	Peer microplanner 30	Rusape	≤24 years	≤49

SHG, self-help group.

### Research team characteristics

Eight trained research assistants (six female and two male) conducted participant recruitment, interviews, translations and transcriptions. Data coding and analysis were performed by a mixed-methods trained researcher with support from four experienced qualitative researchers.

### Data collection

The interview guides were designed using elements adapted from the MRC process evaluation framework, particularly feasibility, acceptability and fidelity.^28 29 31^ Interview guides had similar structure and intent for each participant group (peer microplanners and FSWs) and were tailored to reflect their different roles and experiences within the microplanning process. Peer microplanners were asked questions about their experience delivering the assessment (implementation fidelity), challenges faced (feasibility), and how they or FSWs responded to it (acceptability). FSW interviews explored their understanding of and reaction to the assessment process, what support they received, and what challenges or benefits they perceived to capture the three elements from the FSWs’ perspective. Interviews typically lasted 45–60 min.

Participants received study information before providing informed verbal consent. All interviews were conducted in Shona at a mutually agreed outdoor space or within health facilities. Interviews were audio recorded, then translated as they were directly transcribed into English by the data collection team who were fluent in both Shona and English. The accuracy of the transcripts was checked against the digital recordings; however, transcripts were not returned to participants. Interviews were anonymised and uploaded into NVivo V.12 analysis software.

### Data analysis

Data were analysed using the three preidentified elements: feasibility, acceptability and fidelity.^29 31^ The data were analysed using a framework analytic approach to familiarise with the data, code within the existing structure, and then map and interpret the data.^32 33^ Researchers in this study familiarised themselves with field summaries and analytic memos that were written by the data collection team within a week of data collection to enhance trustworthiness. To support credibility and immersion in the data, the team read and re-read the transcripts before using the three preidentified MRC framework informed elements (feasibility, acceptability and fidelity) to code and re-code the data. Data were then charted and deductively mapped data back to the three elements of interest identifying relationships between themes and subthemes.^32^ This approach enabled interpretation within the selected framework supported by iterative discussion with the data collection team for confirmability.^31 34^ Additionally, reviewing transcripts repeatedly and involving the data collection team members in validating emergent themes and interpretation allowed for reduction of bias and triangulation to confirm whether findings aligned with participants’ realities.^30 34 35^ Reporting of findings was guided by the Consolidated criteria for Reporting Qualitative research guidelines.^36^

## Results

### Themes

**Figure 3:**
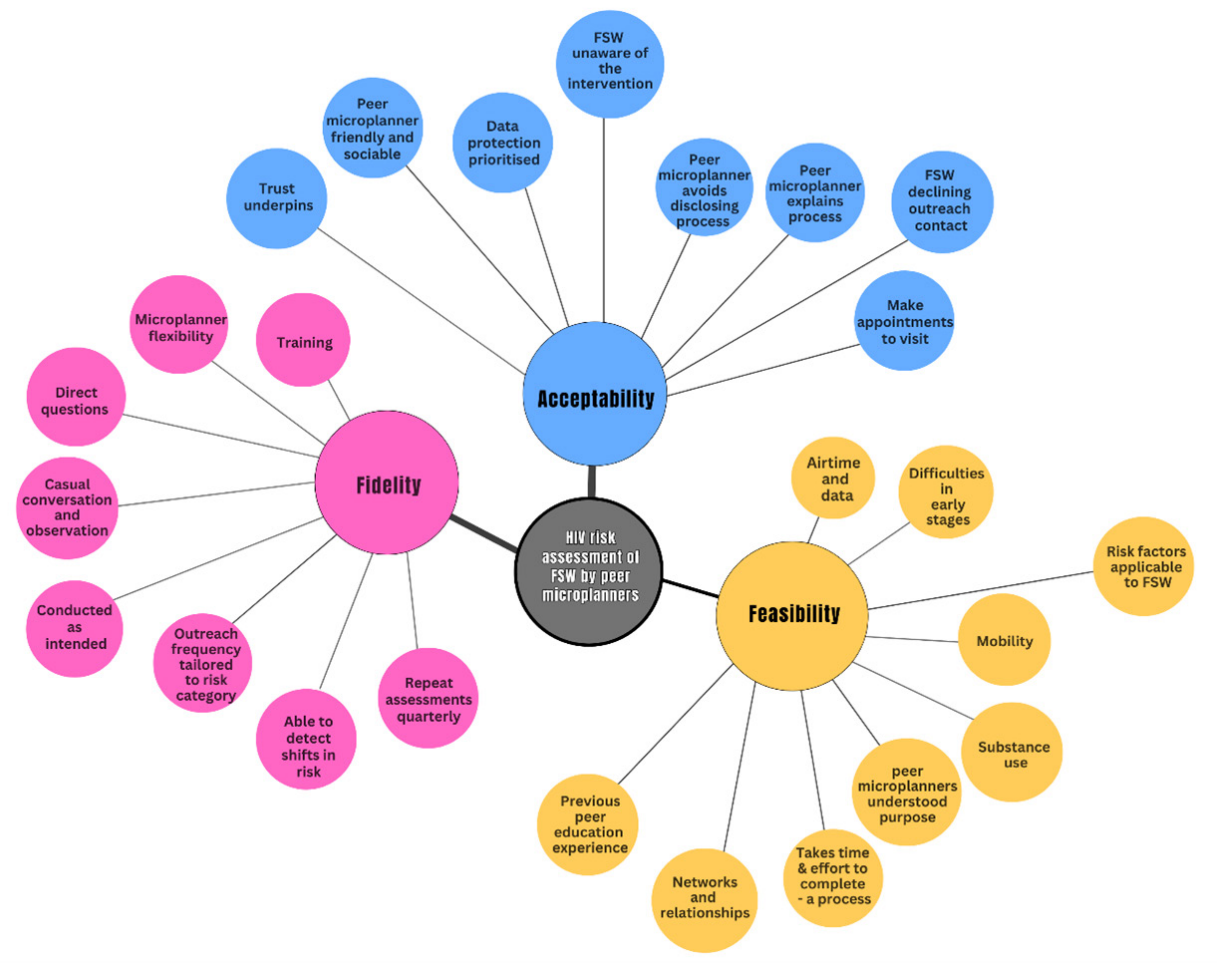
Map of the themes narratively summarised within the preidentified feasibility, acceptability and fidelity elements. FSW, female sex worker.

Figure 3 illustrates the mapping of themes identified to the preidentified elements informed by the MRC process evaluation framework.

### Feasibility

The majority of microplanners understood the purpose of the assessment tool, as demonstrated by 86% (89/104) of peer microplanners who completed risk assessments for >80% of their caseload. Peer microplanners could clearly describe the process and the logic for deciding whether or not each woman was exposed to one of the six factors.

If she is [aged] above 24, I would put zero and if she were below, I would put one. Then I would also look at her period in sex work, if she is new, below 6 months, or above 6 months… I would also ask her if she has more than 10 clients per week and if she says yes, then I would put one and if it’s below 10, I would put zero. I would also ask if there were times when she was not using condoms and if she says yes, I would put one and if there is none, I put zero. In terms of taking alcohol, I would ask if she took drugs and alcohol, but I would respond to this question myself because I actually know who gets intoxicated and who doesn’t. - Microplanner #9, Ngundu

There were some difficulties experienced during the initial period of implementation as peer microplanners worked out how best to approach FSW to collect sensitive information. It was necessary to identify appropriate locations and times better suited to private conversations. In some instances, peer microplanners visited FSW at home. Practice over time improved the ability to undertake assessments.

Long back it was hard for us [doing assessments] but now we are used to it, there is nothing difficult for me now. - Microplanner #3, Chinhoyi

Observations and conversations about risk factors were part of a process that took more than one quarterly contact to determine all the scores. However, the time taken to complete assessments was not reported as cumbersome or overburdening. It was largely integrated into microplanners’ routine interactions with FSW.

I conduct risk assessment after 3 months but on some occasions, I will already know certain things. Like does she cause noise at the bar, or whether she uses a condom or not when she gets drunk. - Microplanner #28, Rusape

Facilitators for completing assessments included good rapport and positive relationships due to pre-existing bonds or efforts to establish strong relationships where these did not already exist.

l must speak with her gently when she responds rudely…l should speak with gentleness so that the person can draw near until l have won her and entered her in my book. – Microplanner #26, Rusape

She and I have a strong link because I share a lot with her. - FSW #28, Rusape

Peer microplanners who had more established networks, either through previous peer education experience or an expansive social network, found microplanning easier, understood principles for establishing rapport better and had a foundation from which to engage women. Existing networks and ability to develop strong new connections appeared particularly valuable, as microplanners who had weaker networks reported struggling with their one-on-one engagements.

Since I was a peer educator before, it wasn’t too difficult for me. - Microplanner #23, Ngundu

I do not have any challenges that I encountered because sex workers in my hotspot are the same people that I relate with…. – Microplanner #12, Rusape

I started off as a microplanner and I was a little confused. It was confusing for me. – Microplanner #30, Rusape

Reasons for peer microplanners being unable to or delaying completion of assessments for some FSWs included FSW mobility or failure to establish a conducive relationship between the microplanner and FSW. Altered moods or behaviour due to substance use also hindered successful outreach contact or assessment of risk.

The FSWs are mobile. I find out that she spent the night in Shongamiti and has not returned yet. By the time I go to her house for the third time and I find out that she is still in Shongamiti and she is there with 5 or 7 other FSWs, because the artisanal miners are there. – Microplanner #21, Ngundu

I don’t stay long in a place when I come back, I may just be around for 2 days and I then leave. - FSW # 26, Rusape

Some will be unavailable and some will be drunk. As you will be addressing those who are drunk it’s just as if you are talking to yourself. There will not be any results and so it means you will have to go back again. – Microplanner #12, Rusape

### Acceptability

Both peer microplanners and FSWs demonstrated that trust underpinned the sharing of information between them. FSWs spoke of sharing personal information with peer microplanners that they would not share with anyone else.

She is the one I talk to when I feel overwhelmed… It’s one person, just one person to whom I tell my secrets. This one who recorded me in her book. – FSW #16, Chinhoyi

She is also a sex worker…there is no way we cannot tell her everything we will be doing. – FSW #2, Chinhoyi

FSWs were more likely to share personal information if they felt their privacy was being respected. Microplanners described creating confidential spaces to help FSW feel comfortable, incorporating assessments into their routine tracking, and being mindful of protecting the data of the FSW they supported to maintain this trust.

She knows that I maintain confidentiality and I will not disclose to anyone. – Microplanner #3, Chinhoyi

She can share with me because it will just be the two of us. – Microplanner #15, Rusape

Despite this trust, divergent views emerged from peer microplanners on how acceptable they felt microplanning, more generally, and assessments of risk specifically, were to FSW. Several peer microplanners did not want FSW to know that data were being collected from them and then used to structure the peer outreach provided. They feared FSW would get angry and violent or would feel they were being ‘exploited’ for the monetary benefit of the monthly allowance paid to peer microplanners for their work.

I do not explain to them that I am tracking them. Because I could be beaten up, because I am making money off them. – Microplanner #13, Rusape

Only a few peer microplanners felt they could be open with FSW about all the details of the intervention. They described ensuring that FSWs fully understood assessments of risk and how these directed the outreach support they received. These peers took time to patiently answer all concerns as part of gaining the FSWs’ trust.

I told them everything including that ‘that the program involves this… depending on the level of risk I need to see you at least once a month if you are at lower risk, if at moderate risk I need to see you twice a month.’ I always made sure I fully explained to them everything. Microplanner #1, Chinhoyi

Though FSWs mostly did not have detailed knowledge of how peer support was structured and delivered, they expressed appreciation for it and for having ready access to the ongoing social and health-related support the peer microplanners provided.

She handles me with care, she does not choose [between us]… I believe that she cares about our lives. – FSW #21, Ngundu

I don’t know whether she is a microplanner or not, I just know that she works for CeSHHAR and I see her whenever I want to see her. A week doesn’t pass without me seeing her. Tracking? I don’t know about it. Risk assessment? I don’t know about it? – FSW #4, Chinhoyi

However, a few FSWs were described as declining outreach contacts, particularly if (1) home visits were too frequent, (2) visits were attempted while a male partner was present, (3) the FSW was intoxicated or (4) the peer microplanner was unskilled in building rapport.

What others don’t like are home visits, it annoys them. When you come one day and then the next, she will feel like you’re suffocating her. – Microplanner #17 Chinhoyi

You just need to be patient. Someone might tell you that she has a client. If she sees you coming through she will just give you a sign that she has a client inside… I will then leave her or I can visit someone else. – Microplanner #7, Ngundu

The programme made some adaptations to facilitate assessments, including providing ‘air time’ to microplanners to enable them to contact their caseload by phone during COVID-19 lockdowns and when travelling. Making an appointment over the phone before visiting women was appreciated by FSWs. It helped facilitate completion of assessments and visits and allowed FSWs to choose the time and place of contact. Peer microplanners stressed the importance of resources being made available to support communication with FSW within their caseloads by phone.

I have to ask for the time of the appointment. Then I tell them I will call them first to find out if they are available before visiting. - Microplanner#13, Rusape

### Fidelity

Assessments of risk were generally conducted as intended, with a median number of 90 days (IQR 89–112) between enrolment and completion of the first assessment. While peer microplanners were clear on what information they needed for women in their caseloads, methods of obtaining the information varied.

Yes, there are FSWs that are below 25 years that are not at higher risk… what I look at other than her age, is the way the FSW conducts her work. The way she sells sex. – Microplanner #6, Ngundu

A few used the assessment tool in its questionnaire format, aided by the worksheets provided, but the majority opted for conversational and observational techniques to determine each woman’s score.

Usually I do an assessment as we will be talking general stories. For example these days I can start talking about Covid effects and from there the conversation goes on. So the approach is different but at the end I know that I might ask the question indirect but all my key areas will be asked. – Microplanner #8, Ngundu

These variations in process did not appear to affect the outcomes and primary purpose. However, how peer microplanners approached the women seemed to have a bearing on whether peer microplanners found completing assessments easy or difficult. To navigate difficult interactions, peer microplanners relied on what they were taught during training.

There are difficult questions like ‘do you use a condom or not’. These are things that may be difficult to ask of which you may not ask directly, but find a polite way. – Microplanner #18, Chinhoyi

During the training we were taught tactics and ways to approach FSWs during risk assessment because some people with whom we work will be drunk and others will be stressed. So because of how we were taught during training, it made me exercise patience with FSWs. So these are the things that made it easy for me to assess a person, I can actually talk to her in a very social way, convincing her to tell me the information that I want. - Microplanner #20, Chinhoyi

Generally, being friendly and sociable, sharing relatable experiences and weaving probing enquiry into daily conversation or activities was described as facilitating the completion of assessments with ease.

If I get to someone’s place and she has not yet cleaned her house, I will help her with cleaning…I can accommodate them… when I am talking to them I will be free, sharing my personal experiences. I will start by sharing my past so that they can understand where I am coming from. – Microplanner #15, Rusape

Several peer microplanners highlighted the need for flexibility and adaptability, especially when approaching FSWs who were irritable or intoxicated. Many microplanners expressed feeling well-equipped for this because of their training.

One challenge that I face is that sometimes when I go for tracking, some of the sisters will be drunk so she may be rude in her response and you should not take it personal. I should not be discouraged…. – Microplanner #16, Chinhoyi

Additionally, they appreciated that experience of risk could change over time and that a change in the frequency of contact in response to changes observed would be required.

Risk can change. It’s a mixed bag, sometimes she may actually improve and then she may get worse. – Microplanner #29, Rusape

Although the group to which someone was assigned guided the frequency of outreach support received, peer microplanners did not always manage to provide outreach contact as frequently as indicated. Overall, peer microplanners delivered an average of 2.5 monthly visits out of an intended 4 for the FSWs at higher risk, 1.5 out of 2 for FSWs at moderate risk, and 1.0 out of 1 for FSWs at lower risk. Peer microplanners felt risk-differentiated microplanning led to improved engagement in services by FSW compared with peer support approach they previously used.

I liked the fact that we are now talking to people individually and you can really understand their challenges. This is different from when we would only talk to people in general. - Microplanner #15, Rusape

Microplanning is [more] powerful than what we did in peer education in the way the girls came to the clinic. It has an impact…you can actually see that there is a big change. - Microplanner #22, Ngundu

## Discussion

This study set out to better understand implementation of assessment of FSWs’ risk of HIV acquisition and transmission by peer microplanners. We found that assessments were implemented with a high level of fidelity, were feasible for peer microplanners to undertake and were largely acceptable to FSW. Trust was important for the process and took time and effort to build. Strong networks and relationships were also crucial.^37^,^39^ This underscores the importance of using a social network mapping tool to identify the most appropriate peer to whom to assign a specific location. Additionally, while it may not be possible to guarantee a peer microplanner has good relationships, capacity gaps were demonstrably addressed during training in this intervention.

An underpinning assumption when designing the intervention was that peer microplanners were uniquely placed to assess FSWs’ exposure to risk factors as they share similar experiences.^37 38^ These proximal relationships gave peer microplanners insights in assessing the HIV risk of FSWs that a service provider or researcher administering a questionnaire would not have. Peer microplanners appreciated the difficulties presented by asking questions about sexual behaviour and the fears that surround the disclosure of such personal information. They were mindful of the need to ensure privacy and confidentiality so that these issues did not hinder FSWs’ participation.

Assessments of risk were easier to complete with time, and ‘teething difficulties’ did not affect overall implementation. There were variable approaches to obtaining the information required to complete assessments. While most peer microplanners relied on observational and conversational methods, a few preferred more direct questions. Ensuring there are structured opportunities for sharing experiences in employing different approaches to obtain the required information was valuable. There was also scope for allowing flexibility and adaptability in implementation while ensuring the oversight by outreach workers continually monitored for functional departures from fidelity.

It is unclear whether those FSWs who were never assessed found it unacceptable (either assessments of risk per se or all of microplanning). It is possible there was insufficient time and number of contacts for assessments to be completed among the unassessed FSWs, the majority of whom were enrolled in the last 9 months of the 30-month intervention. While there were barriers to completing assessments of risk, including mobility, substance use and COVID-19 disruptions, the data suggest these can mostly be overcome through taking time to develop adequate trust and strong relationships.

Although FSWs consented to be listed in the peer microplanner’s hotspot diary and to be supported by them, ethical issues may arise from differentiation of support based on an assessment of need which the individual is unaware of. As described by Reamer (2015),^40^ our study also found that peer microplanners took on and navigated multiple roles while developing informal relationships that at times resembled friendship or transitioned into it.^41^ There may be advantages to the lack of FSW awareness of the assessment, which include reducing chances of social desirability bias and possible tension within FSWs whose experiences remain static despite peer support. However, trust built between the two parties is relied on to access sensitive information.^37^ While peer microplanners in our study endeavoured to maintain confidentiality and avoid harm, due consideration should be given to whether it is contrary to the best interests of the individual to provide them with differentiated support without fully informing them.

The manner in which the research interviewers probed for perspectives on microplanning and assessments during the interviews may have been a weakness as far as obtaining FSW views on the intervention is concerned. As beneficiaries of the intervention, FSW did not use the language of micro-planning and assessment of risk. Future research should further explore these and take care to use language that is relevant to FSWs.

Little was found in the literature on the perspectives and experiences of peer outreach workers and FSWs regarding assessment of risk as part of the peer outreach support provided to FSWs. A review of the literature on peer-led health interventions more broadly showed that peers’ experiences in delivering health interventions are often understudied.^37 38 42^ As recommended in prior research by Simoni *et al*, future studies should further explore how peer outreach workers deliver interventions to gain an understanding of what makes peers effective in that delivery and provide explanations as to why these interventions work well or not.^38 39^

## Conclusions

In this study, we documented how a standardised quantitative tool to assess HIV risk was implemented with fidelity. Although it was not just a quarterly ‘tick box’ exercise, it was feasible for peer microplanners to implement and use to focus peer outreach. It was also acceptable to the FSWs who received support more tailored to their needs as a result. This method of characterising and categorising exposure of FSWs to HIV risk can be integrated into existing work of the peer microplanners. It benefits from the trust established between peer microplanners and individuals in the caseloads they support. Additionally, our study showed that administration of a seemingly straightforward tool involves subtle forms of social interaction over time for completion. However, there remain some gaps in understanding FSW perspectives.

Findings from this study could be used to develop, implement and optimise assessments of risk by peer microplanners in other key population groups and in other contexts. In addition, exploring how to improve the assessment of risk, particularly for mobile individuals, would be of value.

## Supplementary material

10.1136/bmjgh-2024-017968 online supplemental file 1

## Data Availability

Data are available on reasonable request. All data relevant to the study are included in the article or uploaded as supplementary information.
