# Supplementary material for: Feasibility and acceptability of peer-led assessment of HIV risk among female sex workers in Zimbabwe
Source: BMJ Glob Health. 2025 Dec 5;10(12):e017968. doi: 10.1136/bmjgh-2024-017968 (PMC12684197; doi:10.1136/bmjgh-2024-017968)
Supplement: online supplemental file 1 [file bmjgh-10-12-s001.pdf]

Appendix 1: Risk assessment worksheets

Condom Sheet

#	Question	Responses
1a	How many clients do you have per week?	
1b	Of those, how many did you use condoms with?	
2	How many condoms do you need per week?	

Problematic Drinking/Drug Use Sheet

#	Question	Responses
1a	Do you drink alcohol or use/inject drugs?	Yes/No
1b	If yes, how often do you drink alcohol or use/inject drugs?	Daily/Weekly/Monthly
1c	Do you sometimes have sex with clients without a condom because you were drinking or using drugs?	Yes/No
1d	Do you think the way you drink /use drugs ever interfere with your daily activities?	Yes/No

Violence Sheet

#	Questions	Responses
1a	Did you experience any violence last week?<br>Did you experience any violence in the last three (3) months?	Yes/No
1b	If yes, who was it with? (Partner/Client/Friend)	
2a	Did you experience any event/occasion that caused you to be violent?	Yes/No
2b	If yes, who was it with? (Partner/Client/Friend)	
3a	Were you forced to have sex without a condom last week?	Yes/No
3b	Were you forced to engage in any sexual acts that you did not consent to in the last three (3) months?	Yes/No
